# Sensor for Distance Measurement Using Pixel Grey-Level Information

**DOI:** 10.3390/s91108896

**Published:** 2009-11-06

**Authors:** José L. Lázaro, Angel E. Cano, Pedro R. Fernández, Yamilet Pompa

**Affiliations:** 1 Electronics Department, University of Alcalá, Polytechnic School, University Campus, Alcalá de Henares, Madrid 28871, Spain; 2 Telecommunications Department, Oriente University, Av. de las Américas, SN, Santiago de Cuba 90900, Cuba; E-Mail: angel.cano@depeca.uah.es

**Keywords:** distance measurement, radiometry, cameras, calibration, infrared measurements, infrared image sensors

## Abstract

An alternative method for distance measurement is presented, based on a radiometric approach to the image formation process. The proposed methodology uses images from an infrared emitting diode (IRED) to estimate the distance between the camera and the IRED. Camera output grey-level intensities are a function of the accumulated image irradiance, which is also related by inverse distance square law to the distance between the camera and the IRED. Analyzing camera-IRED distance, magnitudes that affected image grey-level intensities, and therefore accumulated image irradiance, were integrated into a differential model which was calibrated and used for distance estimation over a 200 to 600 cm range. In a preliminary model, the camera and the emitter were aligned.

## 1. Introduction

Distance estimation using vision sensors is an important aspect of robotics since many robot positioning algorithms use distance to calculate the robot's position as the basis for more complicated tasks.

Traditionally, distance measuring in robotics has been conducted by sonar (US) and infrared (IR) sensing. Several methods based on the line-of-sight (LOS) and echo/reflection models have also been used. The LOS model places the emitter and detector in different locations, and signals travel from emitter to detector. The Reflection model links emitter and detector physically (in the same place) and signals are reflected off an object or wall, following a round-trip-path.

In the Reflection model, the viability of IR as an accurate means of measuring distance depends on extensive prior knowledge of the surface (scattering, reflection, and absorption). [1] details a method for determining surface properties, and subsequently calculating the distance to the surface and the relative orientation of the surface in an unknown environment using previously acquired sensory data; the developed sensor provides accurate range measurements when used in conjunction with other sensing modalities. In [2], low-cost infrared emitters and detectors are used for the recognition of surfaces with different properties in a location-invariant manner. In order to resolve the dependency of intensity readings on the location and properties of the surface, the use of angular intensity scans and an algorithm to process them was proposed. In [3], an IR sensor based on the light intensity back-scattered from objects and capable of measuring distances, and the sensor model, are described. In all cases [1-3], sensors for short distances are evaluated.

Vision devices based on a geometrical model have been used for many positioning tasks. However, most vision positioning algorithms are based on geometrical imaging models, where 3D to 2D projection constitutes the main mathematical tool for analysis [1-6]. With vision devices, the LOS model for signal transmission and distance measurement is used.

With geometrical models, a single camera and interest point can only estimate a 2D position, as projection based models cannot provide depth information. Nevertheless, depth can be calculated if additional information is included in the model. This entails the use of two vision sensors or some kind of active device [1,2,4,5,7-13].

To date, artificial vision has been one of most widely used positioning techniques since it gives accurate results. However, geometric modeling is more normally used in order to obtain distance to objects [14].

In intelligent spaces, smart living, etc., where a certain number of cameras are already installed, a simple method based on grey levels can be developed to determine the depth. The cameras already installed in the environment are used for performing other tasks necessary in smart living and intelligent spaces. For example, if a mobile robot carries an IRED, depth can be estimated from the cameras' pixel values, because there is a relationship between pixel grey-level intensity and the quantity of light that falls on the image sensor. Also, received light is related by inverse distance square law to the distance between the camera and the IRED.

When an image is captured by a digital camera, it provides a relative measure of the distribution of light within the scene. Thus, pixel grey-level intensities are a function of sensor surface irradiance accumulated during the exposition time.

The function that relates accumulated image irradiance to output grey-level intensities is known as the Radiometrical Camera Response Function (RCRF) [15-18].

In [16], the properties shared by all camera responses were analyzed. This enabled the constraints that any response function must satisfy, and the theoretical space of all possible camera responses, to be determined. Using databases, the authors concluded that real-world responses occupy a small part of the theoretical space of all possible responses. In addition, they developed a low-parameter empirical model of response.

In most cases, an inverse-RCRF is required in order to obtain a direct relationship between pixel grey-level intensities and accumulated image irradiance by a Radiometric Camera Calibration Process (RCCP) [16,17].

Furthermore, if the camera takes an image of an IRED and this can be isolated, a point source model for the IRED can be used to estimate the irradiance on the surface of the camera lens using inverse distance square law [19]. The camera lens captures irradiance distribution on the surface of the sensor and also accumulates sensor irradiance during the exposition time. Finally, the pixel grey-level intensity can be related to the accumulated image irradiance by a RCCP, lens irradiance can be related to the image's sensor irradiance by lens modeling, and lens irradiance can be related to the distance between the camera and the IRED by inverse distance square law. Thus, a relationship with pixel grey-level intensity can be defined which includes the distance between the camera and the IRED.

Previous papers have been presented in the field of IR, in which the authors developed computer programs and methods. In [20], a reducing-difference method for non-polarized IR spectra was described, where the IR signal of a five component mixture was reduced stepwise by subtracting the IR signal of other components until total elimination of the non-desired signals was achieved.

The aim of the present study was to use a geometrical model for 2-D positioning, which would provide coordinates on a plane parallel to a mobile robot (for example), and then to use a radiometrical model in order to obtain the distance between the mobile robot and the plane. For our purposes, the LOS model was used in order to determine the distance between emitter (IR) and detector (CMOS camera).

## 2. The IRED-Camera Set

The method using a single IR point to develop an effective and practical method is based on the assumption that the final application will be carried out in settings such as intelligent spaces, smart living spaces, etc., where a certain number of cameras are already installed. The cameras already installed in the environment are used for performing other tasks necessary in these smart living and intelligent spaces.

One possibility would be to use a photodiode. However, these devices provide an instantaneous response to the signals received, and given the distances involved in these applications (several meters), and working with an LOS link (the best alternative available), these can be less than pW. The low intensity of the received signal can, therefore, impede accurate, or even valid, measuring. Since distance estimation and robot position determination can involve measurements on a ms. timescale, considered as real time, the signal must be integrated into a determined time interval. The use of a photodiode would imply the need to design signal conditioning, integration and digital circuits, etc. All of these, in addition to a webcam, are already available in the proposed method; consequently, the design is simpler, implementation is quicker and final costs are lower, as a variety of existing models can be selected.

A further reason for using a camera is that by applying a differential method for measuring, and given that by using cameras the method can be selected digitally from a computer, automation and control, speed and safety of data acquisition is facilitated since two consecutive measurements are taken with different integration times.

Later, when the method for distance estimation using mathematical algorithms has been fully developed, it will be possible to combine this data with data obtained from the geometric calibration of cameras in order to improve generation of the variables and parameters involved in position and location of the device incorporating the IRED.

**Figure 1.**
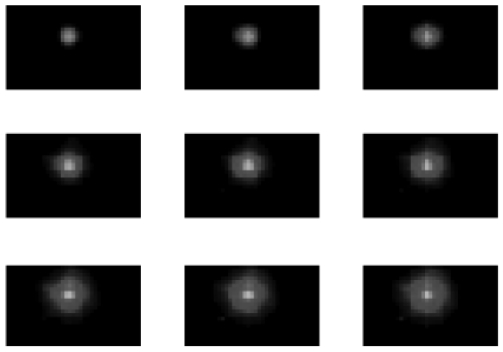
Representative samples of images used in the emitter-to-camera distance characterization.

In order to define a model for the IRED-Camera set, the following aspects were established:

1. Estimation of accumulated image irradiance. When inverse RCRF is used, a measure of the accumulated image irradiance can be obtained. A sample of energy accumulated in the camera is shown in Figure 1.
2. The relationship between accumulated image irradiance and lens irradiance. This can be obtained using the camera's optical system model and also includes the camera exposition time. A linear behaviour is assumed for the camera's optical system model.
3. Behaviour of lens irradiance with the distance between the camera and the emitter. For the IRED Camera set a point source model can be used; in this case, lens irradiance can be estimated using inverse distance square law.
4. Image irradiance must be due to IRED light energy. Therefore, background illumination must be suppressed. An ideal implementation would be to test the algorithm in a dark room; however we used an interference filter to select a narrow wavelength band centered on the typical emitter wavelength in order to ensure that images were created only by IRED energy.

From a general point of view, accumulated image irradiance is a function of camera parameters and radiometric magnitudes which quantitatively affect the image formation process.

The fact that the emitter transmits up to 120°, or at a different angle, only influences the angle at which the detector can be situated in order to receive the emitter's signal, and does not affect the size of the image formed.

As regards image size, according to the laws of optical magnification this is only influenced by the size of the object and the distance at which it is located. In our case, as the diode size is both constant and very small, the image appears reduced. Nevertheless, as can be seen in Figure 1, the point of light image increases in size as the distance of image acquisition decreases.

### 2.1. Model definition

Reference [21] was taken as the starting point, and the inverse RCRF “*g*” was estimated using the method proposed by Grossberg and Nayar [16]. However, a new practical measure for accumulated image irradiance *E_r_* was defined thus:

(1) $$E_{r}=\frac{1}{A}\sum_{i=1}^{A}g(M_{i})$$

where *M_i_* is the normalized grey-level intensity for the pixel *i*, 1≤ *i* ≤ *A*, and where *A* is the total number of pixels in an image's region-of-interest, containing the spot produced by the IRED. In practice, a ROI of 100 pixels × 100 pixels was selected. Since the camera and the IRED were aligned, the same ROI was selected for all the images used.

To define the differential model, the magnitudes and relationships affecting *E_r_* which were defined in [21], were also used here.

A differential method was selected because a measurement taken with a specific exposition time will include various errors due to camera irradiance, external illumination factors (spotlights, the sun, etc.), or the effect of temperature on the sensor, for example. The differential method enabled us to isolate the measurement from these effects.

Moreover, both the sensor and method are economic, since cameras are already installed in the application environment. Furthermore, the method is simple to launch and installation of the system is easy. The system is non-invasive and safe to operate, and the sensorial system is complementary to other methods, facilitating ease of data fusion

## 3. Differential Model

Assuming that the camera and the emitter are aligned initially, there are three magnitudes that affect the accumulated image irradiance for the camera-IRED set the camera exposition time, the IRED radiant intensity and the distance between the IRED and the camera [21].

In addition, and as in [21], the behavior of *E_r_* with each of the magnitudes affecting the IRED image formation process was measured by fixing values for the other magnitudes. For example, in order to discover how *E_r_* behaves with camera exposition time, images were captured using fixed values for the emitter radiant intensity and distance whilst varying the exposition time. A similar methodology was used to obtain all *E_r_*'s behaviors.

As in [21], the same *E_r_* behaviours with defined magnitudes were obtained. Therefore, all measured behaviors could be integrated into a unique expression as follows:

(2) $$E_{r}=(\tau_{1}t+\tau_{2})\times (\rho_{1}P_{e}+\rho_{2})\times \left(\delta_{1}\frac{1}{d^{2}}+\delta_{2}\right)$$

where *τ_1_, τ_2_, δ_1_, δ_2_, ρ_1_*, and *ρ_2_* are the model parameters, and *t, P_e_*, and *d* are the exposition time, the IRED radiant intensity and the distance between the camera and the emitter, respectively.

From (2), this can be re-written as:

(3) $$E_{r}=k_{1}\frac{P_{e}t}{d^{2}}+k_{2}\frac{P_{e}}{d^{2}}+k_{3}\frac{t}{d^{2}}+k_{4}\frac{1}{d^{2}}+k_{5}P_{e}t+k_{6}t+k_{7}P_{e}+k_{8}$$

where *k_j_*, 1≤ *j* ≤ *8*, are model parameters that can be related to *τ_1_, τ_2_, δ_1_, δ_2_, ρ_1_*, and *ρ_2_*. The expression (3) has been obtained by suppressing the parentheses in (2).

If images captured with different camera exposition times are analyzed, then (3) can be written by considering the differences of accumulated image irradiances as follows:

(4) $$E_{r_{\cdot t_{n}}}-E_{r\cdot_{t_{r}}}=k_{1}\frac{P_{e}}{d^{2}}(t_{n}-t_{r})+k_{3}\frac{t_{n}-t_{r}}{d^{2}}+k_{5}P_{e}(t_{n}-t_{r})+k_{6}(t_{n}-t_{r})$$

where *t_n_* and *t_r_* are the different camera exposition times, *t_r_* is the fixed reference exposition time and *tn* represents different exposition time values.

Expression (4) was used as the proposed model to characterize the IRED-Camera set Therefore, values for *k* in (4) must be obtained in a calibration process.

Once *k* parameters have been obtained, the expression (4) can be solved for the distance estimation:

(5) $$d_{n}=\sqrt{\frac{k_{1}P_{e}(t_{n}-t_{r})+k_{3}(t_{n}-t_{r})}{E_{r_{\cdot t_{n}}}-E_{r\cdot_{t_{r}}}-k_{5}P_{e}(t_{n}-t_{r})-k_{6}(t_{n}-t_{r})}}$$

The aim was to use the proposed differential model to estimate the distance between the camera and the IRED, where the model analyzes images of the IRED captured with different exposition times, and also assumes that distance and emitter radiant intensity are constant during the image capturing process.

The method described in this paper, based on differential image processing, presents several advantages, innovations and benefits related to previous studies, which can be summarized as follows:

- the development of a sensor for distance measuring and pose detection based on grey level intensity of the images;
- the development of a method for obtaining the distance between two points (IR-camera) using a differential;
- the sensor and method are economic, since cameras are already in the application environment (requiring only one IRED for each object/mobile to be detected);
- the method is simple to launch;
- installation of the system is easy;
- the system is non-invasive and safe;
- the sensorial system is complementary to other methods, facilitating ease of data fusion; etc

## 4. Practical Implementation

For practical reasons, a Basler camera was used, with a SFH42XX High Powered IRED with 950 nm emission peak; thus an interference filter centered at 950 nm and with a bandwidth of 40 nm was added to the camera, which improved the signal/noise ratio since it eliminated background illumination: all visible light, and infrared light up to 930 nm and over 970 nm.

**Figure 2.**
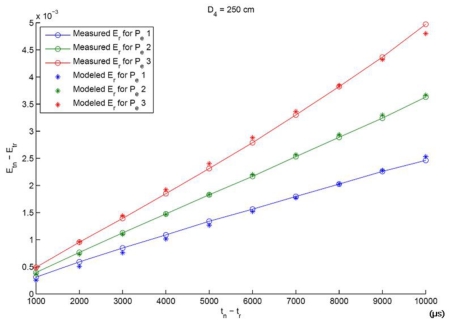
Representative results for the differential model calibration process. Three different emitter radiant intensities were considered by changing the diode polarization current.

In order to use (5) for distance estimation, *k_i_* parameters must be estimated in a calibration process. This process was implemented by analyzing an image sequence of 4 different distances (d1 = 400 cm; d2 = 350 cm; d3 = 300 cm; and d4 = 250 cm), 10 different exposition time differences, assuming *t_r_* = 2 ms as the reference exposition time (the reference exposition time selected was sufficiently low to eliminate possible offset illumination and dark current effects) and *t_n_* = {3; 4; 5; : : : ; 12} ms and 3 different IRED radiant intensities, which were selected by varying the diode's polarization current. A representative result for the calibration process is shown in Figure 2.

In Figure 2, modeled and measured differences of accumulated image irradiances are shown versus exposition time differences. These values were extracted from images used in the calibration process, specifically for the distance *d_4_* = 250 cm, where three different emitter radiant intensities and 10 different exposition time differences were considered. In addition, Figure 2 shows the effectiveness of the model calibration process. Once the calibration process has been carried out, experiments for distance estimation can be conducted.

Two experiments were performed to test the validity of the differential model for distance estimation. In the first experiment, the 200 cm to 380 cm range was considered whilst the second considered the 400 cm to 600 cm range. The second experiment showed greater error since the distances were greater and were also beyond the range for which the sensor had been calibrated (that is, the range used in the first experiment). Nevertheless, the second experiment shows that even so, sufficiently accurate measurements can still be taken. For both experiments, distance was increased stepwise by 20 cm and the camera was aligned with the IRED.

In addition, to improve the efficiency of the methodology in practical applications, four images were analysed to estimate distance. The first image was captured with a *t_r_* = 2 ms of exposition time and the others were captured with *t_1_* = 9 ms, *t_2_* = 10 ms and *t_3_* = 11 ms respectively, thus obtaining three distance estimations. The final distance estimation was the mean value of these distance estimations. The IRED radiant intensity was fixed at *P_e_2* corresponding to a diode polarization current of 5 mA.

## 5. Results

**Figure 3.**
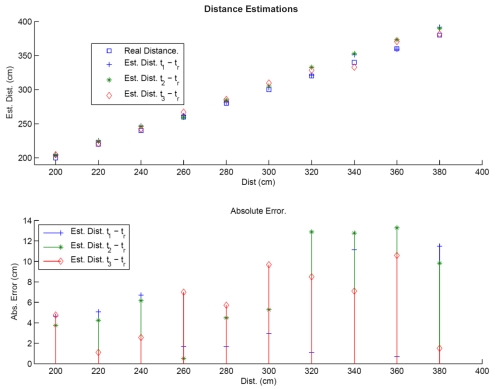
Representative distance estimations and absolute error for Experiment 1.

Experiments were carried out using the method described above. The equipment used is indicated in Section 4. Once images from the optimal exposition time range had been selected, the deferential method for distance estimation was applied. The distance estimation results for each difference in exposition time, corresponding to the first experiment, are shown in Figure 3.

The final distance measurement is shown in Table 1.

In the first distance range, errors in distance estimation using the differential model are less than 8 cm., representing a relative error of less than a 2.5%. The second experiment considered longer distances, and results are given in Table 2.

In this case, the differential model was less accurate than in the first experiment.

## 6. Conclusions

An alternative method based on a radiometrical approach to the image formation process has been described for estimating the distance between a camera and an IRED. This method estimates the inverse RCRF in order to obtain a measure of the accumulated image irradiance, and shows that the accumulated image irradiance depends linearly on the emitter radiant intensity, the camera exposition time and the inverse square distance between the IRED and the camera. These behaviors are incorporated into a model, which can be re-written in a differential form.

The differential model has four parameters that must be estimated by means of a calibration process. Once the model's parameters have been calculated, the model's expression can be solved for distance estimation. Two distance ranges were considered for model validation. In the first range, errors were less than 8 cm. However, in the second experiment the errors were higher than in the first.

In conclusion, the proposed differential model represents an alternative method for estimating the distance between an IRED and a camera through analysis of image grey-level intensities.

## Figures and Tables

**Table 1.** Final distance estimation result for Experiment 1.

**Real Dist. (cm)**	**Est. Dist. (cm)**	**Abs. Err. (cm)**	**Relat. Err. (%)**
200	204.4	4.4	2.2
220	223.5	3.5	1.6
240	245.1	5.1	2.1
260	262.7	2.7	1.0
280	284	4.0	1.4
300	306	6.0	2.0
320	327.5	7.5	2.3
340	345.6	5.6	1.6
360	367.7	7.7	2.1
380	387.6	7.6	2.0

**Table 2.** Final distance estimation result for Experiment 2.

**Real Dist. (cm)**	**Est. Dist. (cm)**	**Abs. Err. (cm)**	**Relat. Err. (%)**
400	412.2	12.2	3.1
420	425.6	5.6	1.3
440	452.6	12.6	2.9
460	487.5	27.5	6.0
480	495.1	15.1	3.1
500	508.3	8.3	1.7
520	543.1	23.1	4.4
540	567.5	27.5	5.1
560	568.0	8.0	1.4
580	595.1	15.1	2.6
600	615.7	15.7	2.6
